# Glucose tolerance in Canadian and French cystic fibrosis adult patients

**DOI:** 10.1038/s41598-019-40592-9

**Published:** 2019-03-18

**Authors:** Quitterie Reynaud, Valérie Boudreau, Sandrine Touzet, Katherine Desjardins, Stéphanie Poupon Bourdy, Emilie Blond, Yves Berthiaume, Rémi Rabasa-Lhoret, Isabelle Durieu

**Affiliations:** 1 Centre de référence Adulte de la Mucoviscidose, Service de médecine interne, Hospices civils de Lyon, F-69495 Pierre Bénite, France; 2 Université de Lyon, Équipe d’Accueil Health Services and Performance Research (HESPER) 7425, F-69003 Lyon, France; 3 Montreal Clinical Research Institute, Québec, Canada; 4 Université de Montréal, Département de nutrition et de Médecine, Montréal, Québec, Canada; 5 Hospices Civils de Lyon, Pôle de Santé Publique, Lyon, F-69003 France; 6 Université de Lyon, Équipe d’Accueil Health Services and Performance Research (HESPER) 7425, Lyon, France; 7 Service de Biochimie et Biologie Moléculaire, Hospices Civils de Lyon, F-69495 Pierre Bénite, France; 8 Université de Lyon, INSERM U1060, Laboratoire CarMen, F-69003 Lyon, France; 9 Cystic fibrosis clinic, Centre Hospitalier de l’Université de Montréal, Montréal, Québec, Canada

## Abstract

Cystic fibrosis (CF)-related diabetes is associated with increased mortality. We analysed the clinical and glycemic profiles of two cohorts of patients treated according to the same guidelines in France and Canada. To investigate incidence differences in phenotypic and glucose abnormalities and to explore the evolution over a 4-year follow-up period, two cohorts of 224 Canadian and 147 French adult CF patients (≥18 years) without treated CF-related diabetes (CFRD) were followed over a 4 year period. In each of these groups, we investigated the longitudinal relationship between glucose tolerance and pulmonary function. An annual 2-hour oral glucose tolerance test was performed: fasting blood glucose (G0) and 2-h blood glucose (G2) were measured. Patients were classified at inclusion according to their glucose tolerance status: Normal glucose tolerant, abnormal glucose tolerant or de novo CFRD. Age, sex ratio and proportion of F508del homozygous patients were not statistically different between both cohorts. Canadian patients had better pulmonary function (median %FEV1 (IQR): 71.0 (55.0–82.0) vs. 64.0 (40.0–78.0), p < 0.001) and greater body mass index (BMI; median BMI in kg/m^2^) (IQR) 21.1 (19.5–22.8) vs. 19.9 (18.4–21.4), p < 0.001). Glucose values: G0 (5.4 (5.0–5.9) vs. 4.8 (4.5–5.1) mmol/L, p < 0.001) and G2 (7.6 (5.8–9.7) vs. 6.5 (5.2–8.5) mmol/L, p = 0.001) were higher in the Canadian cohort translating into a higher incidence of *de novo CFRD* diagnosis (19.2 vs. 9.8%, p = 0.003). Decline in FEV1 over time was not different between patients according to glucose tolerance groups. Despite higher glucose levels and incidence of *de novo* CFRD, Canadian CF patients have a better lung function and a higher BMI than French patients. In spite of these differences between the cohorts, the decline in FEV1 in patients with abnormal glucose tolerance is similar between these groups.

## Introduction

In the last decade, life expectancy of patients with cystic fibrosis (CF) has significantly improved. While the median life expectancy was 10 years of age in the 1960’s, it is now estimated to greater than 50 years both in Canada and France^1,2^. However, along with this better life expectancy, new complications have emerged. Abnormalities in glycemic status and CF-related diabetes (CFRD) have become the main complications after respiratory disease and exocrine pancreatic insufficiency^3^.

The prevalence of CFRD increases with age, affecting around 50% of adult CF patients. In adult patients without CFRD, the prevalence of abnormal glucose tolerance is 35%^4^. A progressive loss of beta cell mass with a direct effect from CFTR-mutation and a possible contributory role of insulin resistance lead to abnormal glucose levels and development of CFRD^5–8^. Because of the insidious onset of CFRD and the fact that standard simple screening tests are less reliable for CF patients (e.g. fasting glucose, glycosylated hemoglobin, etc.)^9^, annual screening for CFRD is recommended starting at ten years of age, using the 2-h Oral Glucose Tolerance Test (OGTT)^10^. CFRD is still associated with increased mortality despite the progressive improvement in screening and management^11^.

Studies have compared demographic data of European and North American CF children patients using CF registries^12,13^, suggesting differences in demographic data (height and weight) between populations. No data are available regarding difference in CFRD prevalence between Europe and North America except data from annual registry reports^14^. Furthermore, registry data concerning specific comorbidities like CFRD are not homogeneously collected, and most of the time registries do not collect data about other glucose metabolic abnormalitites. It is thus possible that both prevalence of CFRD and its impact on clinical CF condition differ between these two populations. The consequences of different glucose tolerance profiles on respiratory function have not been evaluated in large cohorts and over long periods. Similarly, the potential role of insulin deficiency and insulin resistance have not been described and compared in large cohorts^15–17^.

The objective of this study was to compare the following parameters between the Canadian and French cohorts: (1) clinical characteristics, (2) glucose and insulin values as well as CFRD incidence and (3) evolution of pulmonary function over a 4-year follow-up period.

## Methods

### Study population

Data were obtained from the Montreal Cystic fibrosis Cohort (MCFC) for Canada and from the Lyon Cystic Fibrosis Cohort (DIAMUCO) for France. Both research cohorts are investigating mechanisms of glucose intolerance as well as associations between glucose intolerance and clinical outcomes in adult patients with CF. The Montreal Cystic Fibrosis Cohort was established in 2004 and all available data at inclusion from patients included between 2004 to 2016 were considered for this analysis. The DIAMUCO Cohort was established in 2009 and all available data at inlusion from patients included between 2009 to 2012 were considered for this analysis. All available follow-up pulmonary function data were included in both cohorts over a 4-year period.

Informed consents have been obtained for all subjects included. The institutional review board of each participating hospital and research ethics board authorized the cohorts in accordance with the current ethical standards (Comité de Protection des Personnes in France, Comité d’éthique de la recherche in Canada), as well as the French data Protection Agency for DIAMUCO Cohort (Comission Nationale de l’Informatique et des Libertés CNIL).

For both cohorts, all adult patients (>18 years) with CF, pancreatic insufficiency and no previous history of treated CFRD were included. Patients who received a *de novo* CFRD diagnosis during recruitment were however included. Main exclusion criteria were pancreatic sufficiency and previous pulmonary transplantation. In patients with ongoing pregnancy, pulmonary exacerbation or current treatment with medication known to interfere with glucose metabolism (e.g. high dose oral steroids and enteral tube feeding), OGTT was delayed upon resolution of this temporary exclusion factor. OGTT results were accepted in patients taking stable, long-term (≥1 year) low-dose oral corticosteroids (maximal dose of 10 mg prednisone per day). At the time of the OGTT, all patients were clinically stable with no recent (<1 month) pulmonary exacerbation or symptoms of acute infection. Included patients represent around 80% of all patients with CF followed at our respective clinics.

### Clinical and biological data

In both cohorts, a harmonized data collection process was used to extract required information from medical charts at inclusion. This included age, sex, CF related genotype, chronic bacteriological colonization and number of intravenous antibiotics courses in the previous year. Chronic bacteriological colonization was defined as follow: 50% or more of samples being positive for a specific bacteria in the preceding 12 months^18^. Other data such as BMI, FEV1, glycosylated hemoglobin (HbA1c) were also collected at inclusion and then between 12 and 18 months during a 4-year follow-up for FEV1. BMI was calculated using weight in kilograms divided by height in square meter (kg/m^2^). Pulmonary function was measured by spirometry using the forced expiratory volume in 1 second in L (FEV1) using Hankinson 1999 formula for FEV1(%)^19^. HbA1c was measured using HPLC automate variant II (Biorad) in France, and immunotubidimeter (Bayer Health Care diagnosis) in Canada. Both HbA1c are aligned with international standards.

### Oral glucose tolerance test (OGTT)

OGTT was realized at the inclusion in both cohorts and was carried out after an overnight fast. Patients were given glucose (1.75 g per kg bodyweight, maximum 75 g) then plasma glucose was measured at start (G0), 1 hour (G1) and 2 hour (G2)^20^. Patients diagnosed with *de novo* CFRD underwent within 2 months a second OGTT to confirm the diagnosis. Plasma insulin was also measured at start (I0), 1 hour (I1) and 2 hour (I2) by immunoradiometric assay (BI-INS-IRMA, Cisbio Bioassays, France) and measures were centralized in Quebec, to obtain comparable values.

Depending on the results of the OGTT at inclusion, patients were classified into different subgroups of glucose tolerance according to international guidelines^10^: Normal Glucose Tolerance (NGT) (G0 ≤ 7.0 mmol/L and G2 ≤ 7.7 mmol/L), Abnormal Glucose Tolerance (AGT) defined as having either indeterminate glucose tolerance (INDET) (G0 ≤ 7.0 mmol/L and G2 ≤ 7.7 mmol/L, but G1 ≥ 11.1 mmol/L) or impaired glucose tolerance (IGT) (G0 ≤ 7.0 mmol/L and G2 > 7.7 mmol/L but <11.1 mmol/L) or de novo CFRD (G0 > 7.0 mmol/L or G2 ≥ 11.1 mmol/L).

### Statistical methods

Values are expressed as median (interquartile range [IQR]) or percentage, as appropriate. Demographic and clinical data, insulin and glucose values from both cohorts were compared at inclusion of patients of each cohort, using non parametric tests (Chi^2^ or Mann-Whitney tests, as appropriate). Subgroups analyses determined by glucose tolerance subgroup classification were then performed to compare clinical status, glucose and insulin values of both cohorts using non parametric tests (Chi^2^ or Mann-Whitney tests, as appropriate). Correlation analysis between BMI and pulmonary function (FEV1), AUC glycemia and AUC insulin were made with Spearman’s correlation. A linear mixed regression model with random intercept and random slope was fitted to assess the effect of glucose tolerance subgroup at entry in the cohort on the mean slope of FEV1 change over 4 years. Effect of glucose tolerance subgroup was controlled for the cohort and age. Interaction between covariates (cohort, NGT and AGT) and time were tested to characterize differences in longitudinal rates of change. The relationship between FEV1 decline and CFRD subgroup (56 patients) was not analyzed due to the small sample size and no available clinical data in the canadian group since confirmed *de novo* CFRD patients are excluded from the cohort after diagnosis.

Analyses were performed using SPSS software (version 24 by IBM, Chicago, USA) and SAS® software (version 9.4, SAS® Institute Inc., Cary, NC, USA). Area under the curve (AUC) for glucose and insulin was calculated using the software GraphPad Prism (GraphPad Software Inc; CA, USA). A probability value ≤ 0.05 was considered as statistically significant.

## Results

### Characteristics at baseline

Data of 224 Canadian and 147 French patients were included (See Table 1). Demographic and clinical data are detailed in Table 1. No difference of sex ratio, proportion of F508del homozygous patients and age was observed between the 2 cohorts. The clinical status of Canadian group was better with higher BMI (median in kg/m^2^ [IQR]) 21.1 [19.5–22.8] vs. 19.9 [18.4–21.4], p < 0.001 and higher FEV1 (median in % [IQR]) 71.0 [55.0–82.0] vs. 64.0 [40.0–78.0], p < 0.001. Accordingly, a higher proportion of patients with mild to normal predicted FEV1 (>70%) was observed in Canadians cohort (51.3% vs. 38.1%, p = 0.012). The proportion of patients colonized with *Pseudomonas aeruginosa (PA)*, *Burkholderia cepacia*, and *Aspergillus* was not statistically different between both cohorts. There was a higher proportion of patients colonized with *Staphylococcus aureus* (SA) in the French cohort (70.1% vs. 55.8%, p < 0.006). The number of intravenous antibiotics courses in the year preceding the OGTT was not statistically different between the 2 groups.

**Table 1:** Comparison of demographic characteristics and clinical data at inclusion of the Canadian and French patients.

	Canada	France	p value
	N = 224	N = 147	
Gender: woman, %	42.0	43.5	0.764*
Age in year, median (IQR)	22.0 (19.0–28.0)	22.5 (19.0–28.7)	0.831
∆F508 homozygous, %	57.9	55.1	0.818*
%FEV1, median (IQR)	71.0 (55.0–82.0)	64.0 (40.0–78.0)	**0.001**
%FEV1 > 70%, %	51.3	38.1	0.012*
BMI in kg/m^2^, median (IQR)	21.1 (19.5–22.8)	19.9 (18.4–21.4)	**<0.001**
Colonized with P. Aeruginosa, %	74.5	67.3	0.155*
Colonized with B. Cepacia, %	2.8	2.7	0.968*
Colonized with S. Aureus, %	55.8	70.1	0.006*
Colonized with Aspergillus, %	42.3	33.3	0.084
Patients requiring IV antibiotics in the year prior the OGTT, %	41.1	49.0	0.238*
Glycemia G0 in mmol/L, median (IQR)	5.4 (5.0–5.9)	4.8 (4.5–5.1)	**<0.001**
Glycemia G2 in mmol/L, median (IQR)	7.6 (5.8–9.7)	6.5 (5.2–8.5)	**0.001**
AUC Glycemia (G0, G1, G2), median (IQR)	1059.5 (914.9–1239.0)	913.5 (761.2–1043.2)	**<0.001**
NGT, %	36.6	53.4	0.003*
INDET, %	16.5	9.0	
IGT, %	27.7	27.8	
De novo CFRD, %	19.2	9.8	
HbA1c in %, median (IQR)	5.8 (5.5–6.1)	5.7 (5.5–6.0)	0.825
Insulin I0 in μU/dl, median (IQR)	3.8 (2.3–5.7)	3.2 (2.2–5.0)	**0.031**
Insulin I2 in μU/dl, median (IQR)	27.4 (16.6–42.3)	18.1 (11.0–33.0)	**<0.001**
AUC Insulin (I0, I1, I2), median (IQR)	2530.0 (1837.0–3649.0)	1974.0 (1302.0–2910.0)	**<0.001**
Stumvoll Index, median (IQR)	0.096 (0.078–0.111)	0.103 (0.088–0.116)	**<0.001**
HOMA-IR, median (IQR)	0.93 (0.55–1.39)	0.68 (0.47–1.07)	**<0.001**

Bold values represent significant differences. Abbreviations: AGT: Abnormal glucose tolerance (INDET: indeterminate glucose tolerance + IGT: impaired glucose tolerance), AUC: area under the curve, BMI: body mass index, CFRD: cystic fibrosis-related diabetes, CRP: C reactive protein, FEV1: predicted forced expiratory volume in 1 second, G0: plasma glucose measured at start of OGTT, G2: plasma glucose measured at 2 hours of OGTT, HbA1c: glycated hemoglobin, HOMA-IR: Homeostasis model assessment of insulin resistance, IV antibiotics: number of days of intravenous antibiotics in the year of OGTT, NGT: normal glucose tolerance, P. Aeruginosa: *Pseudomonas aeruginosa*, S. Aureus: *Staphylococcus aureus*, *: p value was determined by chi^2^.

The incidence of *de novo CFRD* diagnosis was higher in the Canadian population (19.2% vs. 9.8%, p = 0.003). Canadian cohort displayed a lower proportion of patients with normal glucose tolerance (36.6% vs. 53.4%, p = 0.003). Both fasting (G0) and 2-hours (G2) OGTT values (median in mmol/L [IQR]) were higher in the Canadian cohort: 5.4 [5.0–5.9] vs 4.8 [4.5–5.1], p < 0.001 for G0; 7.6 [5.8–9.7] vs. 6.5 [5.2–8.5], p = 0.001 for G2. The AUC OGTT curve for glucose values is higher in Canadian patients (p < 0.001). However, there was no statistical difference in HbA1c values between the two groups. Regarding insulin values (median μU/dl [IQR]) in Canadian compared to French patients, fasting (I0) and I2 values were higher in the Canadian cohort: 3.8 [2.3–5.7] vs. 3.2 [2.2–5.0], p = 0.031 for I0; and 27.4 [16.6–42.3] vs. 18.1 [11.0–33.0], p < 0.001 for I2. For the entire OGTT test area under the curve for insulin values is higher in Canadian patients (p < 0.001).

### Clinical status comparison among the different glucose tolerance groups

Subgroup analyses determined by glucose tolerance subgroup classification were performed (Table 2). Fourteen French patients were excluded from the analyses because data for either G1 or G2 were not available and their data did not allow classification into glucose tolerance subgroups. %FEV1 (median in % [IQR]) was higher for Canadian patients for the NGT group compared to NGT French patients: 72.0 [56.0–86.0] vs. 64.0[38.0–80.0], p = 0.006, while no difference was observed for %FEV1 between Canada and France for the AGT and CFRD groups. BMI (median in kg/m^2^ [IQR]) was also significantly higher for NGT and AGT Canadian patients compared to the French patients: 20.8 [19.5–22.7] vs. 20.2 [18.4–21.4], p = 0.010 for NGT and 21.1 [19.5–23.1] vs. 19.8 [18.5–21.6], p = 0.002 for AGT.

**Table 2:** Comparison between glucose tolerance groups of the Canadian and French patients.

	NGT			AGT (INDET + IGT)			*De Novo* CFRD		
	Canada	France	P value	Canada	France	P value	Canada	France	P value
n	82	71		99	49		43	13	
Age in year, median (IQR)	22.0 (20.0–25.0)	24.0 (19.0–31.0)	0.063	22.0 (19.0–27.0)	22.0 (19.0–26.0)	0.591	25.0 (20.0–30.0)	27.0 (20.0.−37.0)	0.736
%FEV1, median (IQR)	72.0 (56.0–86.0)	64.0 (38.0–80.0)	**0.006**	73.0 (54.7–82.0)	65.0 (42.5–81.5)	0.143	61.0 (51.0–79.0)	51.0 (42.5–67.0)	0.098
%FEV1 > 70%, %	53.6	39.4	0.079*	56.1	42.8	0.129*	35.7	23.1	0.396*
BMI in kg/m^2^, median (IQR)	20.8 (19.5–22.7)	20.2 (18.4–21.4)	**0.010**	21.1 (19.5–23.1)	19.8 (18.5–21.6)	**0.002**	21.1 (19.3–22.9)	20.2 (18.8–21.1)	0.178
HbA1c in %, median (IQR)	5.7 (5.3–5.9)	5.7 (5.5–6.0)	0.325	5.7 (5.5–6.0)	5.8 (5.5–6.0)	0.759	6.1 (5.8–6.9)	6.3 (5.6–6.7)	0.796
G0 in mmol/L, median (IQR)	5.2 (4.9–5.5)	4.7 (4.4–5.0)	**<0.001**	5.4 (5.0–5.8)	4.8 (4.5–5.2)	**<0.001**	6.3 (5.4–7.4)	5.0 (4.7–6.3)	**0.005**
G2 in mmol/L, median (IQR)	5.6 (4.8–6.7)	5.4 (4.6–6.5)	0.234	8.3 (6.8–9.5)	8.3 (7.6–9.2)	0.851	13.6 (11.5–16.7)	13.0 (12.3–14.2)	0.437
AUC Glycemia (G0, G1, G2), median (IQR)	881 (783–943)	834 (735–918)	**0.005**	1105 (1041–1212)	1026 (951–1122)	**<0.001**	1513 (1342–1776)	1293 (1212–1447)	**0.006**
AUC Insulin (I0, I1, I2), median (IQR)	2483 (1906–3768)	1866 (1257–2649)	**<0.001**	2652 (2022–3678)	2283 (1318–3247)	**0.040**	2008 (1520–3255)	1833 (1083–2856)	0.278
Stumvoll index, median (IQR)	0.110 (0.099–0.117)	0.114 (0.103–0.121)	**0.039**	0.093 (0.085–0.105)	0.092 (0.087–0.104)	0.735	0.060 (0.048–0.071)	0.065 (0.050–0.074)	0.432
HOMA-IR, median (IQR)	0.88 (0.53–1.40)	0.64 (0.39–1.04)	**0.004**	0.91 (0.54–1.32)	0.65 (0.49–1.04)	**0.048**	1.17 (0.69–1.49)	1.00 (0.53–1.32)	0.331

Bold values represent significant differences. Abbreviations: AGT: Abnormal glucose tolerance (INDET: indeterminate glucose tolerance + IGT: impaired glucose tolerance), AUC: area under the curve, BMI: body mass index, CFRD: cystic fibrosis-related diabetes, CRP: C reactive protein,FEV1: predicted forced expiratory volume in 1 second, HbA1c: glycated hemoglobin, HOMA-IR: Homeostasis model assessment of insulin resistance, NGT: normal glucose tolerance. Mann-Whitney analysis were performed except for p value with * that were determined by chi^2^.

#### Glucose values according to glucose tolerance group

For each glucose tolerance category (see Table 2), Canadian patients displayed higher G0 median values and higher glucose median AUC as compared to French patients: p < 0.001 and p = 0.005 in NGT group, p < 0.001 and p < 0.001 in AGT group, and p = 0.005 and p = 0.006 in CFRD group.

#### Insulin values according to glucose tolerance group

NGT and AGT Canadian patients displayed higher insulin median AUC than French patients (p < 0.001 and p = 0.040), but this difference no longer exists for patients with *de novo* CFRD, p = 0.278 (see Table 2).

Insulin sensitivity (Stumvoll index) was higher for NGT French patients (p = 0.039) compared to Canadian NGT patients, but no difference was observed in the 2 other subgroups. Insulin resistance (HOMA-IR) was higher for Canadian NGT and AGT patients (p = 0.004 and p = 0.048) compared to French patients.

**Figure 1:**
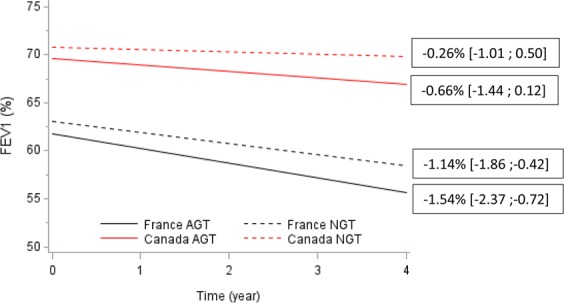
Insulin secretion (μU/dl) at start, 1-h and 2-h of the OGTT for. (**a**) NGT, (**b**) AGT and (**c**) CFRD patients according to their respective cohorts: black dot (•) for Canadian patients and black square (◼) for French patients. Values are presented as mean ± SEM. Abbreviations: AGT: abnormal glucose tolerance, CA: Canadian patients, CFRD: cystic fibrosis-related diabetes, FR: French patients, NGT: normal glucose tolerance.

Despite higher level of insulin values for Canadian patients, the trends of the curve of insulin profile is similar for both Canadian and French NGT and AGT patients. Patients of both cohorts with NGT present a plasma insulin rise during the first hour of the OGTT (Fig. 1a), followed by moderated reduction at 2-h when glucose levels are trending downward. For all AGT patients, a similar insulin profile as for NGT-patients is observed for the 1^st^ hour of the OGTT (Fig. 1b), but then insulin levels remain high at the second hour. For CFRD patients (Fig. 1c), if insulin values are similar at the end of the test for both cohorts, it is slightly higher in Canadian patients at 1-h. However, the 1 h insulin peak observed in NGT and AGT patients is reduced by approximatively 30% for CFRD patients. Insulin values keep rising during the second hour of the test for Canadian and French CFRD patients.

#### Insulin sensitivity and resistance and correlation analysis between BMI and pulmonary function, and between glycemic and clinical parameters in the global cohort

We observe higher insulin sensitivity during the OGTT using the Stumvoll index (median [IQR]) in NGT patients 0.111 [0.102–0.119] vs. both AGT 0.093 [0.085–0.104] and CFRD 0.059 [0.047–0.071] groups and in AGT patients 0.093 [0.085–0.104] vs. CFRD 0.059 [0.047–0.071] (p < 0.001). For fasting insulin resistance (HOMA-IR, median [IQR]), it is higher in CFRD 1.15 [0.71–1.45] than in NGT 0.76 [0.47–1.16] and AGT patients 0.82 [0.51–1.22] (p = 0.013).

**Figure 2:**
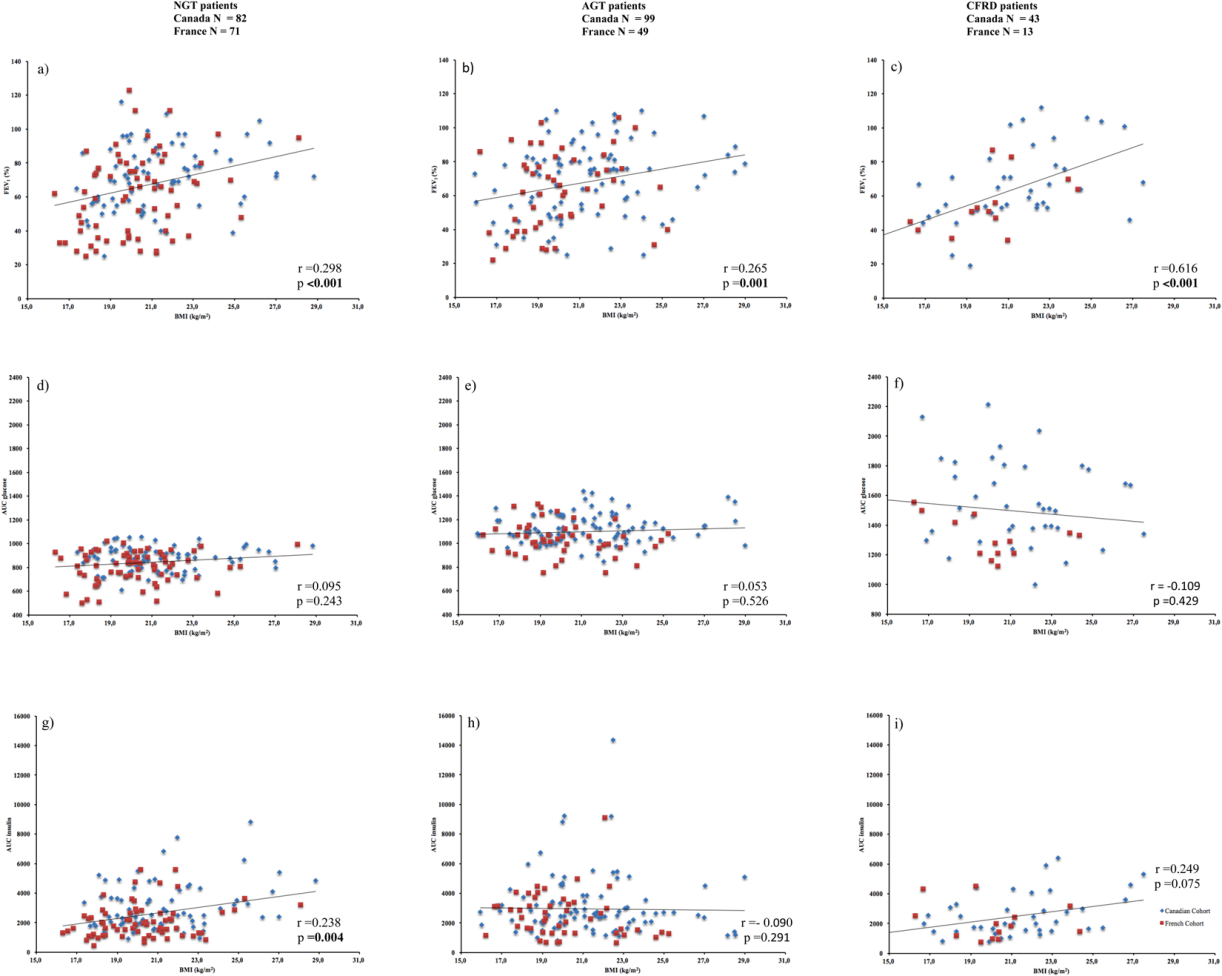
Spearman’s correlation for (**a**) BMI and FEV1in NGT patients (**b**) BMI and FEV1 in AGT patients and (**c**) BMI and FEV1 in CFRD patients (**d**) BMI and AUC glucose in NGT patients (**e**) BMI and AUC glucose in AGT patients (**f**) BMI and AUC glucose in CFRD patients (**g**) BMI and AUC insulin in NGT patients (**h**) BMI and AUC insulin in AGT patients (**i**) BMI and AUC insulin in CFRD patients. Blue diamond: Canadian cohort and Red square: French cohort.

After controlling for cohort, we observed a significant positive correlation (Fig. 2) between BMI and pulmonary function (FEV1) for all glucose tolerance groups (respectively p < 0.001, p = 0.001, p < 0.001 for NGT, AGT and CFRD). No significant correlation was observed for all subgroups between BMI and AUC glycemia. Concerning BMI and AUC insulin, a significant correlation was observed in NGT subgroup (p = 0.004) but not in AGT and CFRD patients. No significant correlation between pulmonary function (FEV1) and AUC glycemia were observed for all glucose tolerance groups (data not shown).

#### FEV1 evolution during follow-up according to glucose tolerance subgroup

**Figure 3:**
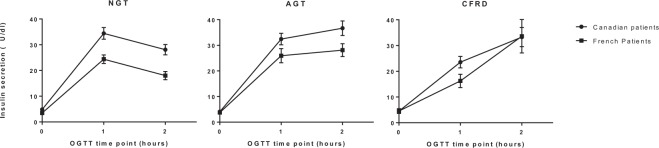
Mean FEV1 change in FEV_1_ according to glucose tolerance subgroup and cohort. Values are presented as mean ± SEM. Abbreviations: AGT: abnormal glucose tolerance, CA: Canadian patients, FR: French patients, NGT: normal glucose tolerance.

FEV1 measurements during the 4-year follow-up period were obtained in 301 patients (81% of overall cohort: 181 French and 120 Canadian). No interaction for glucose tolerance subgroup with time were observed, indicating that the longitudinal changes in FEV1 were not different between NGT and AGT groups (difference in mean annual FEV1 change in NGT compared to AGT group (0.4% 95% CI [−0.5–1.3], p = 0.375). However in all glucose tolerance subgroup a significant difference in mean change in FEV1 per year was observed when French and Canadian patients were compared with Canadian patients having a slower mean annual decline of their FEV1 (difference in mean annual FEV1 change 0.89%, 95% CI [0.0;1.77], p = 0.049), Fig. 3.

## Discussion

To our knowledge, this is the first study that compares the incidence of glucose abnormalities in adult patients with CF, in two large cohorts and their association with clinical status. Despite higher glucose levels and incidence of *de novo* CFRD, Canadian CF patients secrete more insulin and have a better pulmonary and nutritional status according to their FEV1 and BMI than French patients. To explore the mechanisms of these differences, we conducted correlation analyses but we observed no correlation between BMI and glycemia AUC. Canadian patients also have slower annual decrease of their FEV1 than French patients during follow-up. No significant difference was observed for pulmonary function change over time between AGT and NGT patients. These observations challenge the concept of a possible causal role of hyperglycemia and/or hypoinsulinemia favouring clinical status (BMI and/or FEV1) degradation.

In the context of a well-established limited insulin secretion in adult CF patients^21,22^, three key factors could contribute to the development of hyperglycemia: progression of insulin secretory deficiency, higher insulin resistance or higher insulin requirements^22,23^. In Canadian patients, we observe higher insulin secretory capacity, higher estimated insulin resistance but also higher CFRD incidence. Recently, insulin resistance variations has emerged as a possible contributor to hyperglycemia for patients with CF^8,24^. Higher BMI could contribute to higher insulin resistance in Canadian patients^25,26^. As insulin is an anabolic hormone, as long as a certain degree of insulin secretion is preserved, this could allow a higher BMI^27^. Indeed, when insulin secretion further deteriorates leading to *de novo* CFRD, both cohorts do not present anymore differences for BMI and insulin secretion. The frequency of exacerbations which is a good marker of respiratory function stability does not seem to be implicated in observed insulin resistance differences as the number of antibiotic courses is similar in both cohorts. It is also possible that insulin secretion itself might explain clinical differences between cohorts rather than blood glucose. A higher insulin secretion level might allow to reach and/or maintain a higher weight^28^. In a context of a limited insulin secretion capacity, hyperglycemia could also play a role by favoring pulmonary exacerbations^29^ and promoting oxidative stress^30^. Both higher insulin as well as lower glucose values can thus contribute to better lung function.

In contrast to previous reports, our results highlight that despite higher glucose values, Canadian CF patients have better pulmonary function and BMI. Despite these higher glucose values, Canadian patients also presented a slower annual FEV1 decline. When both cohorts are combined and the pulmonary evolution of patients with AGT is compared to patients with NGT, there is no difference in annual FEV1 decline. Various factors not related to cystic fibrosis can also explain this observation. Among them, the role of genetic factors other than CFTR mutations may explain the observed differences between these two cohorts (modifier genes). In the present study, the distribution for F508del CFTR mutation proportions is similar between the two cohorts but it is now well established that other mutations are associated, for some of them, with lung disease severity and for others, with diabetes susceptibility^31,32^. This emerging important factor may also have a role in observed glucose and BMI differences. For example, some mutations in genes involved in a higher risk for type 2 diabetes (e.g. TCF7L2) are also associated with a higher and earlier CFRD prevalence^32^. Further investigations need to be done to compare modifier genes between the 2 cohorts. Secondly, despite comparable nutritional and clinical recommendations for CF care as well as health care systems (universal access) between the 2 cohorts, other factors can still influence glucose values as well as the nutritional status of CF-patients. For example, differences in qualitative and quantitative nutritional intake could impact both glucose tolerance and BMI. Unfortunately, nutritional intake was not assessed in the present study. If done in the future, such assessment could be limited by the precision of available tools (e.g. food journals, 24-hour dietary recall, etc.) as well as the fact that most tools are country specific thus limiting the ability to compare different populations^33^. In addition, despite merging two large and well characterized cohorts, the sample size of some subgroups, such as de novo CFRD, remains small thus limiting our ability to explore some differences.

In order to interpret our results, the potential mechanisms of higher BMI in Canadian patients should be explored. Key factors involved in energy balance and nutritional intake, absorption and energy expenditure (physical activity, energy demand related to CF exacerbations, etc.) should be evaluated. Nutritional recommendations are similar in North America and Europe with a recommended energy intake range from 120 to 150% of energy needs for the healthy population of similar age, sex and size. Patients included in both countries are also exposed to similar pancreatic enzyme replacement therapy (PERT) protocol, starting at 500 U lipase/kg/meal to a maximal dose of 1000–2500 U lipase/kg/meal which should lead to similar nutrient absorption capacity. Thus the two cohorts should be exposed to similar quantitative nutritional intake and absorption. The frequency of exacerbations necessitating intravenous antibiotics does not seem to play a role on BMI values as the number of antibiotics course is similar in both cohorts. International guidelines for antibiotic use in CF are worldwide applied and this may contribute to the very close use of antibiotics in Canadian and French cohorts. However, chronic higher caloric intake and/or differences in physical activity may still be important factors in explaining the differences and may play a role in the higher BMI of Canadian patients. In addition, backgroud population differences in diabetes and obesity, which are both higher in Canada compared to France^34–37^, could also explain the disparities between French and Canadians independently of CF status. As previously reported herein, there is a positive correlation between BMI and FEV1 which could explain the higher FEV1 observed in Canadian patients as well as their lower mean annual FEV1 decline.

Observed association and differences do not imply causality and despite our careful assessement of two large and well characterized cohorts important underlying mechanistic factors were not measured in that study.

In conclusion, Canadian patients present a better clinical status (higher BMI, insulin secretion and FEV1) than French patients, but unexpectedly they also present a higher incidence of glucose abnormalities. In addition, patients in the abnormal glucose tolerance group do not have worse mean FEV1 decline over observed time than patients with normal glucose tolerance. To better understand the complex interplay between glucose tolerance and clincal status (BMI and/or FEV1) of adult patients with CF, further investigations should focus on potential underlying factors that may play a role in the observed differences.
